# Validation of a Commercially Available Enzyme ImmunoAssay for the Determination of Oxytocin in Plasma Samples from Seven Domestic Animal Species

**DOI:** 10.3389/fnins.2017.00524

**Published:** 2017-09-21

**Authors:** Cecile Bienboire-Frosini, Camille Chabaud, Alessandro Cozzi, Elisa Codecasa, Patrick Pageat

**Affiliations:** Research Institute in Semiochemistry and Applied Ethology Apt, France

**Keywords:** oxytocin, measurement, enzyme immunoassay, analytical validation, extraction, pets, farm animals, mammals

## Abstract

The neurohormone oxytocin (OT) has a broad range of behavioral effects in mammals. It modulates a multitude of social behaviors, e.g., affiliative and sexual interactions. Consequently, the OT role in various animal species is increasingly explored. However, several issues have been raised regarding the peripheral OT measurement. Indeed, various methods have been described, leading to assay discrepancies and inconsistent results. This highlights the need for a recognized and reliable method to measure peripheral OT. Our aim was to validate a method combining a pre-extraction step, previously demonstrated as essential by several authors, and a commercially available enzyme immunoassay (EIA) for OT measurement, using plasma from seven domestic species (cat, dog, horse, cow, pig, sheep, and goat). The Oxytocin EIA kit (EnzoLifeSciences) was used to assay the solid-phase extracted samples following the manufacturer's instructions with slight modifications. For all species except dogs and cats, concentration factors were applied to work above the kit's sensitivity (15 pg/ml). To validate the method, the following performance characteristics were evaluated using Validation Samples (VS) at various concentrations in each species: extraction efficiency via spiking tests and intra- and inter-assay precision, allowing for the calculation of total errors. Parallelism studies to assess matrix effects could not be performed because of too low basal concentrations. Quantification ranges and associated precision profiles were established to account for the various OT plasma concentrations in each species. According to guidelines for bioanalytical validation of immunoassays, the measurements were sufficiently precise and accurate in each species to achieve a total error ≤30% in each VS sample. In each species, the inter-assay precision after 3 runs was acceptable, except in low concentration samples. The linearity under dilution of dogs and cats' samples was verified. Although matrix effects assessments are lacking, our results indicate that OT plasma levels can reliably be measured in several domestic animal species by the method described here. Studies involving samples with low OT plasma concentrations should pay attention to reproducibility issues. This work opens new perspectives to reliably study peripheral OT in a substantial number of domestic animal species in various behavioral contexts.

## Introduction

Oxytocin (OT) is a 9-amino-acid neurohormone that is universally present in mammals and plays a key role in regulating a broad variety of social behaviors ranging from social memory and affiliation (sexual and parental behaviors, attachment, pair bonding) to aggression (“mate-guarding,” maternal aggression) (for reviews, see Lim and Young, 2006; Lee et al., 2009; Ziegler and Crockford, 2017). Emotional behaviors, such as anxiety-related behaviors and stress coping are also modulated by OT (Uvnäs-Moberg, 1998; Amico et al., 2008; Neumann, 2008). In particular, OT has been described as being involved in positive social interactions in intra-species interactions (Grewen et al., 2005; Baumgartner et al., 2008; Ditzen et al., 2009; Dunbar, 2010; Smith and Wang, 2012; Romero et al., 2015; Numan and Young, 2016) and inter-species interactions, such as human-animal interactions (Odendaal and Meintjes, 2003; Beetz et al., 2012; Rault, 2012, 2016; Romero et al., 2014). Some authors even suggest a role of OT in enhancing well-being (Uvnäs-Moberg and Petersson, 2005; Ishak et al., 2011).

Consequently, there is a growing interest in investigating and explaining the role of OT in behavioral neuroscience, which has led to an increasing number of studies on the topic. Some of these studies have focused on behavioral effects in different contexts of OT administration. Routes for OT administration are variable: of note, the intranasal administration of OT has recently solicited considerable interest because of its non-invasive nature, its ease of use, and its direct access to the brain, circumventing the blood-brain barrier and the rapid degradation of OT in blood (Veening and Olivier, 2013; for reviews, see Olff et al., 2013). Importantly, Lee et al. (2017) developed a method based on spectrometry to distinguish endogenous and exogenous OT that showed that intravenously or intranasally administrated OT were able to reach the brain in a similar way. Another approach investigating the behavioral/emotional effects of OT is based on correlational studies measuring OT levels in biological samples, such as excretory fluids (urine, saliva), blood or cerebrospinal fluid (Alves et al., 2015). However, the assessment and interpretation of urine and saliva measurements provide inconsistent findings and require further investigation, notably using efficient assay methods (Horvat-Gordon et al., 2005; Anderson, 2006; Carter et al., 2007; Young and Anderson, 2010). Conversely, plasma and CSF samples, despite being more invasive (though to a lesser extent for plasma), displayed interesting results with established correlations between OT concentrations in CSF and plasma samples in several species (Neumann et al., 2013; Carson et al., 2014; Dal Monte et al., 2014). Of note, Dal Monte et al. (2014) and Freeman et al. (2016) obtained contradictory results regarding the correlation of OT concentration in CSF and plasma in the same species *Macaca mulatta*, which can be explained by looking more closely at the measurement methods used: Dal Monte et al. (2014) used extracted plasma to assay OT and found correlation between OT levels in plasma and CSF while (Freeman et al., 2016) used unextracted plasma and did not find any correlation.

Indeed, there are several methodological issues regarding OT measurement reliability since the methods used to analyze plasma OT greatly vary in published reports that investigate the role of OT in social behavior, leading to some inconsistent results. The assay methods described in these studies vary widely and may or may not apply plasma extraction (with different types of extraction protocols), as previously highlighted (Christensen et al., 2014; Robinson et al., 2014). Historically, radioimmunoassay (RIA) procedures were used, usually involving prior extraction procedures (Kendrick et al., 1991; Gilbert et al., 1996; Wotjak et al., 1998; Cool and Debrosse, 2003; Handler et al., 2003). Little by little, enzyme immunoassays became more prevalent than RIA due to their safety and ease of use, and for unknown reasons, extraction steps were also gradually abandoned by some researchers as highlighted by Dal Monte et al. (2014), McCullough et al. (2013), and Nave et al. (2015). But, since the beginning of enzyme immunoassay (EIA) use to assay OT in plasma, some authors have added methodological caveats after having firmly demonstrated that extraction was crucial in order to remove interfering immunoreactive molecules and obtain consistent OT concentrations with the first studies involving RIA (Szeto et al., 2011; Robinson et al., 2014). Notably, in an elegant study, Szeto et al. (2011) proved the necessity of performing extraction both in RIA and EIA to eliminate compounds that artificially increase the apparent plasma OT levels. Indeed, they demonstrated that the linearity and the accuracy of measurements was only verified in extracted samples and that there was no correlation between OT values in extracted samples and OT values in unextracted samples. Additionally, using HPLC characterization of immunoreactive fractions, they showed that some non-OT immunoreactive products (with molecular masses larger than that of OT) persist even in the extracted samples. The authors suggest that these products could come from OT degradation. The critical importance of extracting plasma to assay OT in order to obtain reliable results was again underlined in various reviews (McCullough et al., 2013; Leng and Ludwig, 2016), and even by the EIA manufacturer (ENZO Life Sciences, 2015).

Furthermore, immunoassays require validation as highlighted and defined by Andreasson et al. (2015), DeSilva et al. (2012), and Kelley and DeSilva (2007). Specifically, the European Medicines Agency (EMA) set up guidelines for bioanalytical validation in 2011 (European Medicines Agency, 2011) to facilitate the adoption of these good analytical practices. In human pharmaceutical research, this approach is now standard. However, this is not necessarily the case when it comes to animal science. In addition, some immunoassay kits designed for use with human samples are directly used with animal samples without prior validation (Young and Anderson, 2010; McCullough et al., 2013). This can be problematic since basal levels of analyte may differ between species and may be subject to matrix effects due to the variable presence of interfering substances in biological fluids. It is then important to validate methods of measurement, even pre-existing or commercial methods, when changing the sample's origin and/or nature to obtain reliable results (Kelley and DeSilva, 2007; European Medicines Agency, 2011). The procedure for validating the method can be adapted according to the application and the intended use of the measured analyte (Lee et al., 2006; Valentin et al., 2011).

The goal of this study is to provide a detailed analytical validation of a reliable and universal method for assaying OT in plasma samples from various domestic animal species. Indeed, in some domestic species, such as pets and farm animals, plasma is the biological fluid most readily available, unlike CSF (which is assessed more easily in studies involving lab animals like mice or rats). The panel of domestic species was chosen to meet the needs that may exist in behavioral/emotional studies involving OT, notably on human-animal interactions (Coulon et al., 2012; Rault et al., 2013; Kis et al., 2015; Oliva et al., 2015; Thielke and Udell, 2017), maternal behavior (Castrén et al., 1993; Hernandez et al., 2002; Poindron et al., 2007; Ishii et al., 2008), or stress coping (Yayou et al., 2010; Sutherland and Tops, 2014), where OT can be assessed as a biomarker for a particular behavioral or emotional state. For the reasons stated earlier, we decided to perform an analytical validation based on the EMA guidelines for OT measurement using extracted plasma samples followed by EIA.

## Materiels and methods

### Study animals

Seven adult mammal species of both sexes were involved in this study. Dogs (*Canis familiaris*), cats (*Felis domesticus*), horses (*Equus caballus*), pigs (*Sus scrofa*), goats (*Capra hircus*), and sheep (*Ovis aries*) were from IRSEA breeding (Apt, France, approval n° A 84-400-1). Cows (*Bos taurus*) were from the experimental farm of the Institut Agricole Régional (I.A.R., Reg. La Rochère 1/A, 11100 Aoste, Italy). The housing, husbandry and the use of animals in the procedures described in this article were carried out following the French and European legislation and in compliance with the principles of replacement, reduction and refinement. All procedures were performed with approval from the Ethics Committee C2EA125, in harmony with French and European legislation.

### Blood sampling

During the morning (7:00–13:00), blood was collected by a veterinarian from the jugular vein of animals involved in this study in pre-chilled EDTA-Aprotinin tubes (BD® tubes, Elvetec, Pusignan, France) and centrifuged at 4°C at 1,200 g to recover plasma. The volume of blood drawn varied according to species. In cases of delay (max. 3 h) between the sampling and centrifugation, tubes were kept at 4°C in order to avoid OT degradation, which was also enhanced by the use of aprotinin as a protease inhibitor. Recovered plasma was aliquoted in several tubes to avoid repeated freeze/thaw cycles, which could degrade OT, and stored at −20°C until further use. To limit OT degradation during the storage, all samples were assayed within 2 months (Marnet et al., 1994).

### Oxytocin enzyme ImmunoAssay

Plasma OT was measured using the EIA kit initially developed by Assay Designs Inc. and currently provided by Enzolifesciences (Villeurbanne, France, catalog No. 900-153A), following the manufacturer's instructions. This kit has been increasingly used in studies assaying OT in various animal species in the last decade (Robinson et al., 2014; Leng and Ludwig, 2016). Notably, it was previously assessed by Szeto et al. (2011) in a thorough work studying the effect of OT extraction from plasma prior to RIA and EIA. According to the manufacturer, the kit's sensitivity is 15.0 pg/ml; intra-assay precision at low, medium and high concentrations are 12.6, 10.2, and 13.3% respectively; inter-assay precision at low, medium and high concentrations are 20.9, 16.5, and 11.8% respectively.

### Plasma oxytocin extraction and concentration

Plasma OT was measured after a solid-phase extraction (SPE) using C18 columns following the manufacturer's instructions with the following modifications. The C18 columns used were 1 g HyperSep (Thermofischer, Illkirch, France) to ensure their capacity to deal with large volumes of plasma and so 2 ml of acetonitrile was used to equilibrate them. The elution buffer was 60% acetonitrile/40% TFA 0.1% since during prior observations on several animals' plasmas, this elution buffer displayed better recovery than the elution buffer recommended by the provider (95% acetonitrile/5% TFA 0.1%). The samples were dried overnight at room temperature in a centrifugal concentrator under vacuum. A volume of 600 μl of Assay buffer was used to reconstitute the samples in order to run the assay in triplicates and to avoid pipetting insoluble material which may be observed in some samples (here, it was particularly the case for dog samples). Before carefully pipetting the reconstituted samples into the EIA plate wells, tubes were also quickly spun to move the insoluble material downward. SPE is strongly recommended by the kit provider (ENZO Life Sciences, 2015). The extraction helps eliminate potential interfering molecules and diminishes the matrix effect. In addition, during the extraction step, OT molecules can be concentrated from the sample prior to EIA for aid in measurement. In this study, sample concentration was necessary for cow (20-times), horse (15-times), pig (10-times), goat (10-times), and sheep (10-times) samples to reach concentration values within the kit's standard range. Dog and cat samples were not concentrated. Consequently, the volume of extracted plasma from each species was adjusted from the beginning of the procedure so that the volume of assay buffer could be held constant at 600 μl and the concentration factors specified above could be applied (for instance, the volume of treated pig plasma was 6 ml in these conditions).

### Assessment of validation criteria

According to the EMA Guideline on bioanalytical method validation (2011), commercial kits need to be revalidated to ensure that the sample analysis is performed accurately and precisely. Additionally, a change of biological matrix or species is a reason to perform a partial validation, which “can range from the determination of the within-run precision and accuracy to an almost full validation.” The precision of an analytical procedure is defined as “the closeness of agreement between a series of measurements obtained under the prescribed conditions” and is expressed as the ratio of standard deviation/mean (%), also known as coefficient of variation (CV%). Two types of precision can be evaluated: the intra-assay precision or within-run precision and the inter-assay precision or between-run precision. The accuracy of an analytical procedure is defined as “the closeness of the determined value to the value which is accepted either as a conventional true value or an accepted true value.” In accordance with EMA recommendations and the principle of the Fit-for-Purpose method validation for biomarker measurements (Lee et al., 2006; Valentin et al., 2011), the authors have chosen to perform a partial validation of the Enzolifesciences Oxytocin EIA kit, including assessment of within-run and between-run precision and accuracy. Of note, parallelism studies to detect possible matrix effects or differing antibodies' affinities between the endogenous analyte and the standard, that assess serially diluted study samples along the standard curve, are also a crucial step of the validation procedure (European Medicines Agency, 2011; Valentin et al., 2011; DeSilva et al., 2012; Andreasson et al., 2015). Regrettably, in view of the low basal levels of plasma OT eventually found in each species, it was not possible to conduct this test since, as soon as the actual samples were diluted, the authors faced the issue of an OT level decrease below the kit's sensitivity. For the assessment of precision and accuracy, three validation samples (VS), which are spiked plasma samples at three levels of concentration, were prepared by spiking study samples with 50 pg of OT standard before the SPE. Thanks to the high concentration of the OT standard (10,000 pg/ml), we were able to ensure the spiking solution represented <5% of the final volume, as recommended by Valentin et al. (2011). They were stored and treated according to the same procedures used for the analysis of study samples, including the extraction and concentration steps, so that the global procedure could be evaluated and validated. It is precisely because this is the global procedure (including possible concentration, extraction and assay steps) which is assessed through the VS spiked at the beginning of the process that the term “accuracy” is now replaced by “extraction efficiency” when specifically relating to the study outcomes, in order to take into account the combination of the SPE efficiency and the proper accuracy of the EIA, which cannot be distinguished in this case. Study samples being run in triplicate, the within-run precision was determined using 3 replicates of measures. Three independent runs were performed on different days, using two different lots of EIA kits from Enzolifesciences, to establish the between-run precision (the kit lot used in run 3 was different from the kit lot used in run 1 and 2). The VS levels of concentration were chosen according to the range of quantification preliminarily established for each species, including at least one level close to the Lower Limit of Quantification (LLOQ) observed within the study samples. The LLOQ is defined as the lowest concentration in a sample which can be quantitatively determined with acceptable levels of precision and accuracy and is different from the Limit of Detection (LOD), or sensitivity, which is defined by the manufacturer in a sample-independent manner. To preliminarily determine the range of quantification for each species, numerous study samples were assayed in triplicate during multiple runs and a precision profile was established, from which the LLOQ and ULOQ (Upper Limit of Quantification) were determined. In this context and in order to stay in close touch with the real samples, all values found along the standard range described by the manufacturer were taken into account, including the ones found above the 80% intercept. Acceptance criteria were as defined by the EMA, as follows: regarding extraction efficiency, the absolute mean bias % relative error (RE) should be ≤20% of the nominal value at each concentration level (≤25% at the interval between 1x and 3xLLOQ) and the precision should not exceed 20% of CV (25% at the interval between 1x and 3xLLOQ). Furthermore, the total error is a concept that expresses the closeness of agreement between a measured test result and its theoretical true value (DeSilva et al., 2012). The term total error describes the combination of systematic error (mean bias) and random error (precision): it is calculated by summing the absolute value of the % RE and the % CV and should not exceed 30% (40% at the interval between 1x and 3xLLOQ). At least 67% of VS samples should reach these acceptance criteria. In addition, the linearity under dilution was assessed in samples where no concentration was required (i.e., dogs and cats' samples) by spiking two pools of plasma samples with two different high amounts of OT standards (800 and 500 pg) to ensure reaching high concentrations within the kit's dynamic range and supporting five points of 1:2 serial dilutions.

## Results

### Precision profiles

**Figure 1:**
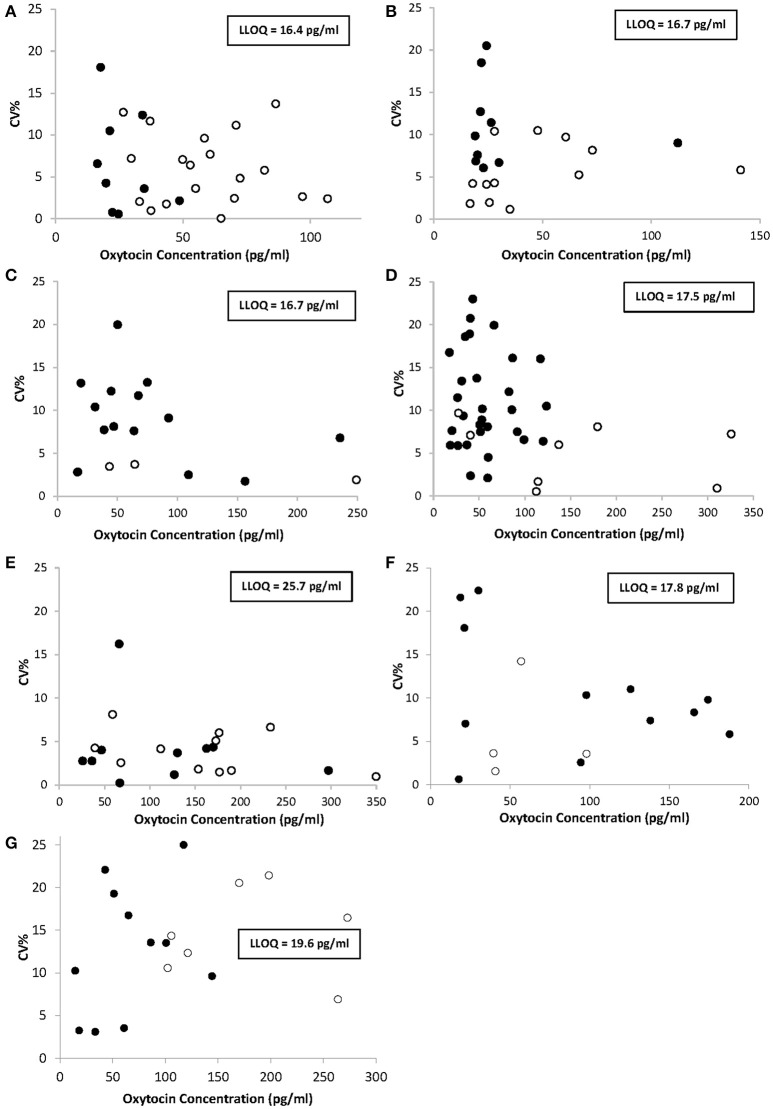
Precision profiles of oxytocin concentrations determined from triplicate measures of multiple runs in neat samples (•) or spiked samples (◦) from dogs **(A)**, cats **(B)**, horses **(C)**, cows **(D)**, sheep **(E)**, goats **(F)**, and pigs **(G)**. %CV indicates the percent coefficient of variation. Oxytocin concentrations stand for the concentrations measured in the wells of the EIA plate and not in the animals' plasma, for which specified concentration factors must be applied. LLOQ, lower limit of quantification.

The precision profiles obtained for each of the seven studied species are presented in Figure 1 and contain values from neat and spiked samples tested throughout the study. Spiked samples served to extend the range of quantification with higher OT concentrations than neat samples. The range of quantification in this study was delimited by the LLOQ determined for each species and shown in boxes inside the charts. Importantly, precision was satisfactory in all the samples tested for each species throughout the quantification range with *CV* <20%, or <25% for concentrations comprised in an interval of 1xLLOQ-3xLLOQ. Dog and cat samples were not concentrated and consequently displayed narrower ranges of quantification (0–150 pg/ml) than other animal samples whose concentrations were multiplied by at least a factor of 10 during the extraction step, leading to a range of quantification extending to a maximum of 350 pg/ml in sheep. When the specified concentration factors are applied, mean plasma OT concentrations can be calculated from neat samples in each species population: 26.6 ± 10.5 pg/ml in dogs (*n* = 9); 10.9 ± 5.0 pg/ml in cats (*n* = 10); 4.6 ± 3.9 pg/ml in horses (*n* = 14); 2.9 ± 1.5 pg/ml in cows (*n* = 30); 11.3 ± 9.0 pg/ml in sheep (*n* = 10); 9.1 ± 6.7 pg/ml in goats (*n* = 12); and 6.7 ± 4.1 pg/ml in pigs (*n* = 11).

### Within-run precision, extraction efficiency and total error assessment

According to the precision profile of each species, VS with OT concentrations falling within the range of quantification determined here were tested to assess within-run precision and extraction efficiency (Tables 1–7) and calculate the total errors. All three dog, cat, cow, sheep, goat and pig VS displayed satisfactory results, with a total error ≤30% and intermediate levels of precision and extraction efficiency with acceptable values, except VS2 in dog and VS3 in cow. In horse VS, VS2 exhibited a total error >30% although intermediate levels of within-run precision and extraction efficiency were acceptable on their own, while VS1 and VS3 gave satisfactory results. Overall, 100% of VS from dogs, cats, cows, sheep, pigs and goats and 67% of VS from horses reached the acceptance criteria.

**Table 1:** Within-run precision, extraction efficiency and total error in dog VS.

	**Precision**			**Extraction efficiency**						**± Total error (%)**	**Total error acceptance criteria (%)**
	**Mean concentration (pg/ml)**	**Within-run precision (%CV)**	**Precision acceptance criteria (%)**	**Expected concentration after spiking (pg/ml)**	**Mean of measured concentration after spiking (pg/ml)**	**Within-run precision (%CV)**	**Precision acceptance criteria (%)**	**% Spiking recovery**	**± Relative error (%)**		
VS1	17.6	18.1	≤25	67.6	54.9	3.6	≤20	81	19	22.6	≤30
VS2	22.2	0.8	≤25	55.5	70.1	2.5	≤20	126	**26**	28.5	≤30
VS3	48.6	2.2	≤25	90.1	72.4	4.9	≤20	80	20	24.9	≤30

*Acceptance criteria in this species were as follows: the absolute mean bias % relative error (RE) and the %CV should be ≤ 20% of the nominal value at each concentration level (≤25% at the interval between 1x and 3xLLOQ, i.e., 16.4–49.2 pg/ml). The total error should not exceed 30% (40% at the interval around the LLOQ). Values out of the acceptance range are indicated in bold*.

**Table 2:** Within-run precision, extraction efficiency and total error in cat VS.

	**Precision**			**Extraction efficiency**						**± Total error (%)**	**Total error acceptance criteria (%)**
	**Mean concentration (pg/ml)**	**Within-run precision (%CV)**	**Precision acceptance criteria (%)**	**Expected concentration after spiking (pg/ml)**	**Mean of measured concentration after spiking (pg/ml)**	**Within-run precision (%CV)**	**Precision acceptance criteria (%)**	**% Spiking recovery**	**± Relative error (%)**		
VS1	21.8	18.5	≤25	41.8	47.8	10.5	≤20	114	14	24.5	≤40
VS2	26.4	11.4	≤25	45.6	34.9	1.2	≤25	77	23	24.2	≤40
VS3	112.2	9.0	≤20	145.2	141.1	5.8	≤20	97	3	8.8	≤30

*Acceptance criteria in this species were as follows: the absolute mean bias % relative error (RE) and the %CV should be ≤ 20% of the nominal value at each concentration level (≤25% at the interval between 1x and 3xLLOQ, i.e., 16.7–50.1 pg/ml). The total error should not exceed 30% (40% at the interval around the LLOQ)*.

**Table 3:** Within-run precision, extraction efficiency and total error in horse VS.

	**Precision**			**Extraction efficiency**						**± Total error (%)**	**Total error acceptance criteria (%)**
	**Mean concentration (pg/ml)**	**Within-run precision (%CV)**	**Precision acceptance criteria (%)**	**Expected concentration after spiking (pg/ml)**	**Mean of measured concentration after spiking (pg/ml)**	**Within-run precision (%CV)**	**Precision acceptance criteria (%)**	**% Spiking recovery**	**± Relative error (%)**		
VS1	17.0	1.1	≤25	79.5	64.4	3.7	≤20	81	19	22.7	≤30
VS2	50.1	3.3	≤25	158.1	128.1	14.9	≤20	81	19	**33.9**	≤30
VS3	235.5	15.7	≤20	297.9	249.1	1.9	≤20	84	16	17.9	≤30

*Acceptance criteria in this species were as follows: the absolute mean bias % relative error (RE) and the %CV should be ≤ 20% of the nominal value at each concentration level (≤25% at the interval between 1x and 3xLLOQ, i.e., 16.7–50.1 pg/ml). The total error should not exceed 30% (40% at the interval around the LLOQ). Values out of the acceptance range are indicated in bold*.

**Table 4:** Within-run precision, extraction efficiency and total error in cow VS.

	**Precision**			**Extraction efficiency**						**± Total error (%)**	**Acceptance criteria (%)**
	**Mean concentration (pg/ml)**	**Within-run precision (%CV)**	**Precision acceptance criteria (%)**	**Expected concentration after spiking (pg/ml)**	**Mean of measured concentration after spiking (pg/ml)**	**Within-run precision (%CV)**	**Precision acceptance criteria (%)**	**% Spiking recovery**	**± Relative error (%)**		
VS1	26.9	1.3	≤25	43.7	40.4	7.1	≤25	92	8	15.1	≤40
VS2	59.9	3.0	≤20	126.6	137.1	6.0	≤20	108	8	14.0	≤30
VS3	91.8	4.6	≤20	154.3	114.5	1.7	≤20	74	**26**	27.7	≤30

*Acceptance criteria in this species were as follows: the absolute mean bias % relative error (RE) and the %CV should be ≤ 20% of the nominal value at each concentration level (≤25% at the interval between 1x and 3xLLOQ, i.e., 17.5–52.5 pg/ml). The total error should not exceed 30% (40% at the interval around the LLOQ). Values out of the acceptance range are indicated in bold*.

**Table 5:** Within-run precision, extraction efficiency and total error in sheep VS.

	**Precision**			**Extraction efficiency**						**± Total error (%)**	**Acceptance criteria (%)**
	**Mean concentration (pg/ml)**	**Within-run precision (%CV)**	**Precision acceptance criteria (%)**	**Expected concentration after spiking (pg/ml)**	**Mean of measured concentration after spiking (pg/ml)**	**Within-run precision (%CV)**	**Precision acceptance criteria (%)**	**% Spiking recovery**	**± Relative error (%)**		
VS1	65.9	16.2	≤25	128.4	111.8	4.2	≤20	87	13	17.2	≤30
VS2	130.3	3.7	≤20	189.7	176.3	6.0	≤20	93	7	13.0	≤30
VS3	297.0	1.7	≤20	356.3	349.5	1.0	≤20	98	2	3.0	≤30

*Acceptance criteria in this species were as follows: the absolute mean bias % relative error (RE) and the %CV should be ≤ 20% of the nominal value at each concentration level (≤25% at the interval between 1x and 3xLLOQ, i.e., 25.7–77.1 pg/ml)*.

**Table 6:** Within-run precision, extraction efficiency and total error in goat VS.

	**Precision**			**Extraction efficiency**						**± Total error (%)**	**Acceptance criteria (%)**
	**Mean concentration (pg/ml)**	**Within-run precision (%CV)**	**Precision acceptance criteria (%)**	**Expected concentration after spiking (pg/ml)**	**Mean of measured concentration after spiking (pg/ml)**	**Within-run precision (%CV)**	**Precision acceptance criteria (%)**	**% Spiking recovery**	**± Relative error (%)**		
VS1	21.5	18.4	≤25	48.1	39.5	3.6	≤25	82	18	21.6	≤40
VS2	23.5	17.2	≤25	50.1	40.7	1.6	≤25	81	19	20.6	≤40
VS3	34.0	11.0	≤25	96.4	97.9	3.6	≤20	102	2	5.6	≤30

*Acceptance criteria in this species were as follows: the absolute mean bias % relative error (RE) and the %CV should be ≤ 20% of the nominal value at each concentration level (≤25% at the interval between 1x and 3xLLOQ, i.e., 17.8–53.4 pg/ml)*.

**Table 7:** Within-run precision, extraction efficiency and total error in pig VS.

	**Precision**			**Extraction efficiency**						**± Total error (%)**	**Acceptance criteria (%)**
	**Mean concentration (pg/ml)**	**Within-run precision (%CV)**	**Precision acceptance criteria (%)**	**Expected concentration after spiking (pg/ml)**	**Mean of measured concentration after spiking (pg/ml)**	**Within-run precision (%CV)**	**Precision acceptance criteria (%)**	**% Spiking recovery**	**± Relative error (%)**		
VS1	19.6	6.0	≤25	86.2	89.6	6.3	≤20	104	4	10.3	≤30
VS2	34.6	1.4	≤25	101.2	99.9	1.5	≤20	126	1	2.5	≤30
VS3	52.3	0.9	≤25	119.0	134.1	7.3	≤20	113	113	20.3	≤30

*Acceptance criteria in this species were as follows: the absolute mean bias % relative error (RE) and the %CV should be ≤20% of the nominal value at each concentration level (≤25% at the interval between 1x and 3xLLOQ, i.e., 19.6–58.8 pg/ml)*.

### Between-run precision

As shown in Tables 8–14, in each species, one VS did not provide satisfactory results regarding between-run precision and this is almost always observed in the VS displaying the lowest concentration (except in pigs' VS), and is sometimes even comprised within the 1x-3xLLOQ interval. Some intermediate levels of within-run precision were also unsatisfactory, particularly in run 2. Of note, results obtained in run 3, from a different kit lot, were not remarkably different from those obtained in runs 1 and 2. Finally, in all species, 67% of VS reached the acceptance criteria for between-run precision.

**Table 8:** Between-run precision in dog VS, established from three independent runs on different days, using two different lots of Enzolifesciences EIA kits.

	**Run 1**			**Run 2**			**Run 3**			**Between-run mean concentration (pg/ml)**	**Between-run precision (%CV)**	**Precision acceptance criteria (%)**
	**Mean concentration (pg/ml)**	**Within-run precision (%CV)**	**Precision acceptance criteria (%)**	**Mean concentration (pg/ml)**	**Within-run precision (%CV)**	**Precision acceptance criteria (%)**	**Mean concentration (pg/ml)**	**Within-run precision (%CV)**	**Precision acceptance criteria (%)**			
VS A	26.1	3.7	≤25	39.7	**31.9**	≤25	52.7	**23.0**	≤20	39.5	**33.7**	≤25
VS B	73.5	10.5	≤20	67.3	**23.8**	≤20	79.9	**27.7**	≤20	73.5	8.6	≤20
VS C	132.8	20.0	≤20	124.7	**24.0**	≤20	125.9	14.3	≤20	127.8	3.4	≤20

*Values out of the acceptance range (i.e., if CV >20%, or >25% at the interval between 1x and 3xLLOQ) are indicated in bold*.

**Table 9:** Between-run precision in cat VS, established from three independent runs on different days, using two different lots of Enzolifesciences EIA kits (EIA kit lot used in run 3 was different from EIA kit lot used in run 1 and 2).

	**Run 1**			**Run 2**			**Run 3**			**Between-run mean concentration (pg/ml)**	**Between-run precision (%CV)**	**Precision acceptance criteria (%)**
	**Mean concentration (pg/ml)**	**Within-run precision (%CV)**	**Precision acceptance criteria (%)**	**Mean concentration (pg/ml)**	**Within-run precision (%CV)**	**Precision acceptance criteria (%)**	**Mean concentration (pg/ml)**	**Within-run precision (%CV)**	**Precision acceptance criteria (%)**			
VS A	361.2	17.6	≤20	338.2	9.1	≤20	312.6	9.3	≤20	337.4	7.2	≤20
VS B	220.8	6.9	≤20	191.0	8.4	≤20	196.4	17.2	≤20	202.8	7.8	≤20
VS C	43.0	24.7	≤25	50.2	**22.8**	≤20	66.0	**21.0**	≤20	53.0	**22.2**	≤20

*Values out of the acceptance range (i.e., if CV >20%, or >25% at the interval between 1x and 3xLLOQ) are indicated in bold*.

**Table 10:** Between-run precision in horse VS, established from three independent runs on different days, using two different lots of Enzolifesciences EIA kits (EIA kit lot used in run 3 was different from EIA kit lot used in run 1 and 2).

	**Run 1**			**Run 2**			**Run 3**			**Between-run mean concentration (pg/ml)**	**Between-run precision (%CV)**	**Precision acceptance criteria (%)**
	**Mean concentration (pg/ml)**	**Within-run precision (%CV)**	**Precision acceptance criteria (%)**	**Mean concentration (pg/ml)**	**Within-run precision (%CV)**	**Precision acceptance criteria (%)**	**Mean concentration (pg/ml)**	**Within-run precision (%CV)**	**Precision acceptance criteria (%)**			
VS A	26.1	3.7	≤25	39.7	**31.9**	≤25	52.7	**23.0**	≤20	39.5	**33.7**	≤25
VS B	73.5	10.5	≤20	67.3	**23.8**	≤20	79.9	**27.7**	≤20	73.5	8.6	≤20
VS C	132.8	20.0	≤20	124.7	**24.0**	≤20	125.9	14.3	≤20	127.8	3.4	≤20

*Values out of the acceptance range (i.e., if CV >20%, or >25% at the interval between 1x and 3xLLOQ) are indicated in bold*.

**Table 11:** Between-run precision in cow VS, established from three independent runs on different days, using two different lots of Enzolifesciences EIA kits (EIA kit lot used in run 3 was different from EIA kit lot used in run 1 and 2).

	**Run 1**			**Run 2**			**Run 3**			**Between-run mean concentration (pg/ml)**	**Between-run precision (%CV)**	**Precision acceptance criteria (%)**
	**Mean concentration (pg/ml)**	**Within-run precision (%CV)**	**Precision acceptance criteria (%)**	**Mean concentration (pg/ml)**	**Within-run precision (%CV)**	**Precision acceptance criteria (%)**	**Mean concentration (pg/ml)**	**Within-run precision (%CV)**	**Precision acceptance criteria (%)**			
VS A	64.5	**31.9**	≤20	67.2	19.5	≤20	101.3	**32.7**	≤20	77.7	**26.4**	≤20
VS B	251.5	**27.3**	≤20	294.8	10.9	≤20	277.7	15.2	≤20	274.7	7.9	≤20
VS C	268.5	12.8	≤20	210.4	19.5	≤20	246.7	18.9	≤20	241.9	12.1	≤20

*Values out of the acceptance range (i.e., if CV >20%, or >25% at the interval between 1x and 3xLLOQ) are indicated in bold*.

**Table 12:** Between-run precision in sheep VS, established from three independent runs on different days, using two different lots of Enzolifesciences EIA kits (EIA kit lot used in run 3 was different from EIA kit lot used in run 1 and 2).

	**Run 1**			**Run 2**			**Run 3**			**Between-run mean concentration (pg/ml)**	**Between-run precision (%CV)**	**Precision acceptance criteria (%)**
	**Mean concentration (pg/ml)**	**Within-run precision (%CV)**	**Precision acceptance criteria (%)**	**Mean concentration (pg/ml)**	**Within-run precision (%CV)**	**Precision acceptance criteria (%)**	**Mean concentration (pg/ml)**	**Within-run precision (%CV)**	**Precision acceptance criteria (%)**			
VS A	425.1	9.2	≤20	419.1	11.4	≤20	386.2	13.4	≤20	410.1	5.1	≤25
VS B	88.9	**32.9**	≤20	77.2	**45.3**	≤20	119.8	**41.6**	≤20	95.3	**23.1**	≤20
VS C	137.1	10.1	≤20	146.6	**37.9**	≤20	170.1	**34.0**	≤20	151.3	11.2	≤20

*Values out of the acceptance range (i.e., if CV >20%, or >25% at the interval between 1x and 3xLLOQ) are indicated in bold*.

**Table 13:** Between-run precision in goat VS, established from three independent runs on different days, using two different lots of Enzolifesciences EIA kits (EIA kit lot used in run 3 was different from EIA kit lot used in run 1 and 2).

	**Run 1**			**Run 2**			**Run 3**			**Between-run mean concentration (pg/ml)**	**Between-run precision (%CV)**	**Precision acceptance criteria (%)**
	**Mean concentration (pg/ml)**	**Within-run precision (%CV)**	**Precision acceptance criteria (%)**	**Mean concentration (pg/ml)**	**Within-run precision (%CV)**	**Precision acceptance criteria (%)**	**Mean concentration (pg/ml)**	**Within-run precision (%CV)**	**Precision acceptance criteria (%)**			
VS A	246.5	15.7	≤20	219.6	36.5	≤20	244.2	31.2	≤20	236.7	6.3	≤20
VS B	127.2	14.2	≤20	126.3	**23.8**	≤20	162.6	**31.6**	≤20	138.7	14.9	≤20
VS C	43.2	**38.0**	≤25	61.5	**52.5**	≤20	71.2	**28.8**	≤20	58.6	**24.2**	≤20

*Values out of the acceptance range (i.e., if CV >20%, or >25% at the interval between 1x and 3xLLOQ) are indicated in bold*.

**Table 14:** Between-run precision in pig VS, established from three independent runs on different days, using two different lots of Enzolifesciences EIA kits (EIA kit lot used in run 3 was different from EIA kit lot used in run 1 and 2).

	**Run 1**			**Run 2**			**Run 3**			**Between-run mean concentration (pg/ml)**	**Between-run precision (%CV)**	**Precision acceptance criteria (%)**
	**Mean concentration (pg/ml)**	**Within-run precision (%CV)**	**Precision acceptance criteria (%)**	**Mean concentration (pg/ml)**	**Within-run precision (%CV)**	**Precision acceptance criteria (%)**	**Mean concentration (pg/ml)**	**Within-run precision (%CV)**	**Precision acceptance criteria (%)**			
VS A	494.8	25.3	≤20	408.2	9.6	≤20	566.0	25.4	≤20	498.7	16.1	≤20
VS B	63.8	**40.9**	≤20	57.9	11.9	≤25	95.6	**3.8**	≤20	72.4	**28.0**	≤20
VS C	58.5	11.8	≤25	75.5	**31.9**	≤20	72.4	**28.0**	≤20	66.3	13.0	≤20

*Values out of the acceptance range (i.e., if CV >20%, or >25% at the interval between 1x and 3xLLOQ) are indicated in bold*.

### Dilutional linearity

**Figure 2:**
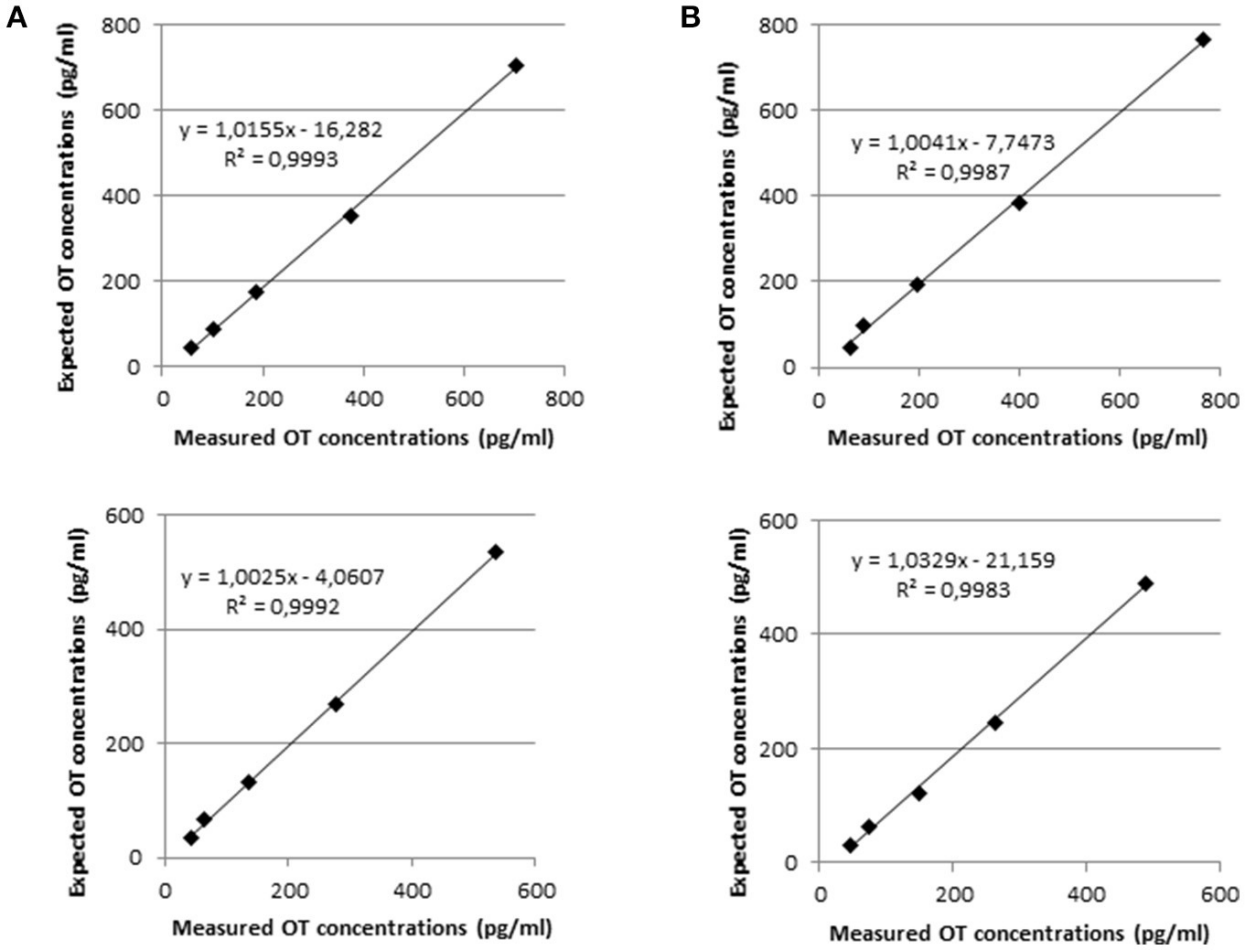
Linearity under dilution of two samples of canine plasma **(A)** and feline plasma **(B)**. The above and below panels show the linearity under dilution was demonstrated in samples spiked with 800 and 500 pg of OT standards, respectively.

In Figure 2, the dilutional linearity was shown in canine and feline plasma samples by reporting the measured values vs. the expected theoretical values, calculated on the basis of the spike amount in the initial samples and the following serial dilutions. The resemblance of the measured and expected values of OT concentration was demonstrated by the slope of the linear regression curve, which is close to 1 in every case. In addition, the coefficient of correlation R^2^ of the linear regression curve was also meaningful as its value nearby 1 showed the concentrations of the diluted samples were not scattered but along the linear regression curve.

## Discussion

In this study, we assessed the suitability of an EIA kit (from Enzolifesciences) for accurately and precisely assaying OT concentrations in solid-phase extracted plasmas from seven domestic animal species. As results obtained in at least 67% of the tested VS were satisfactory regarding the acceptance criteria established in agreement with the EMA Guidelines and the Fit-For-Purpose principle (Lee et al., 2006) for within-run precision and extraction efficiency, we can conclude that the use of this method has been validated for the measurement of plasma OT levels in dog, cat, horse, cow, sheep, goat and pig samples. Furthermore, 100% of VS fulfilled all acceptance criteria in cats, sheep, goats, and pigs. Between-run precisions were also satisfactory and yet highlighted substantial variations in the measurements near the LLOQ values. This was observed in almost all studied species and consequently the authors recommend cautiously interpreting the results obtained in these lowest ranges of concentrations, especially when several runs are needed. For this reason, and because of the sensitivity limit of the kit at 15 pg/ml, we decided to perform sample concentration during the extraction steps in almost all species, except for dogs and cats for which it was not necessary to do so. Indeed, in these species, the basal OT values found in this study were above the kit's LOD. For the other species, assay attempts were made without concentrating their plasma, but were unsuccessful as we found out afterwards that their basal peripheral levels of OT were below the kit LOD. Sample concentration was thus necessary to reach OT values in the dynamic range of the EIA kit and concentration factors were adapted to each species after several analyses. Nevertheless, sample concentration required higher amounts of plasma, and thus a greater volume of blood, which must be drawn without jeopardizing animal health. This has to be taken into account from an ethical point of view and considering the 3Rs rule (Franco and Olsson, 2014). In the case of domestic animals enrolled in this study, they were of sufficient size to safely supply the volume of blood required.

Here, the authors have chosen to perform a partial validation since the kit was of a commercial nature and already validated for use in human plasma. Indeed, the EMA Guidelines state that a full validation is not required when a change in biological matrix occurs, which is the case in this study, with plasma originating from domestic animals. A partial validation can range from as little as the determination of the within-run precision and accuracy to a nearly full validation. In our partial validation, we established the precision profile of OT measurements in each studied species and assessed the extraction efficiency (reflecting the global procedure accuracy as explained before), within-run and between-run precisions. Importantly, despite precision and extraction efficiency yielded satisfactory results, we were not able to assess matrix effects. Even if the SPE method and the use of assay buffer as diluent to reconstitute samples are expected to remove interfering substances and minimize the risk of matrix effects (Tate and Ward, 2004), we cannot be sure that all potentially interfering substances are indeed removed, especially after concentration of the plasma samples. So, results should still be interpreted with caution, as perhaps discrepancies might be found because of putative persisting matrix effects in some cases. Despite their importance, parallelism studies between the calibration standard curve and serially diluted study samples could not be performed due to very low basal levels of OT in each species, which made it impossible to obtain the needed serial dilutions for this kind of study, even after concentrating the samples many times. As already stated, the sample concentration required a large amount of plasma to be taken, which was a real constraint depending on the studied species, notably in the smallest animals. In addition, handling large volumes of plasma is a source of increasing errors and practical complications entailed with the use of HyperSep C18 columns, which can clog. So further increasing the concentration factors to artificially elevate the sample OT concentrations in order to carry out parallelism studies was not a desirable option, considering that it would also have alienated the aim from using real samples reflecting the actual study populations, as recommended by Valentin et al. (2011). Finally, a parallelism study can also provide doubtful evidence, as highlighted by McCullough et al. (2013) about the results obtained without extraction steps in Kramer et al. (2004), which were subsequently questioned by Szeto et al. (2011). Besides, because of the prior extraction steps, we did not purposely include lipemic or hemolytic samples in our study. Indeed, in a series of experiments, we observed that extraction steps were effective enough to prevent any discrepancies in the measurements. In the present study, dilution was never required, and samples from 5/7 species even had to be concentrated. However, to provide data on the resemblance between the endogenous analyte and the standard, we performed dilutional linearity tests on dogs and cats' samples, where no concentration was required. The linearity under dilution was ascertained in both samples origin, throughout the linear range of the kit. Despite the differences of biological matrix and the putative outcomes, it is also interesting to note that the dilutional linearity was demonstrated by the manufacturer in human serum (ENZO Life Sciences, 2015). As stated by Lee et al. (2006), the proven dilutional linearity could support parallelism.

Furthermore, the fit-for-purpose principle states that “assay validation should be tailored to meet the intended purpose of the biomarker study, with a level of rigor commensurate with the intended use of data” (Lee et al., 2006). In the present context of research on the link between OT and multiple behaviors or emotional status in domestic animals, OT can be considered as an exploratory biomarker. This is why the analytical validation performed here comprised 3 VS levels, as well as 3 independent runs with 3 replicates per run for precision and accuracy assessment, as supported by the literature (Lee et al., 2006; Valentin et al., 2011). In addition, we chose triplicate analysis over the “classical” duplicate analysis since we observed a significant within-run variability in some cases (DeSilva et al., 2012). In particular, we noticed that insoluble material after sample reconstitution in assay buffer could heavily influence the calculated concentrations, which may be falsely overestimated due to a decrease in OD in the wells where insoluble material has been observed throughout the EIA procedure. The presence of insoluble material was especially noticeable in dog plasma samples and proved very difficult to remove. To resolve this issue, we added a step to the procedure for all species; samples were spun briefly after the reconstitution in assay buffer and were then carefully pipetted into the plate wells.

Interestingly, the precision profiles of neat samples showed that the basal plasma levels of OT observed in every species involved in this study were coherent with those previously described in the literature. For dogs, when samples were extracted, OT mean concentrations using RIA were found to be approximately 63 pg/ml in bitches in anestrus (Olsson et al., 2003) and 45 ± 40 or 65 ± 82 pg/ml (in two different breed populations) using the same EIA method (Hollinshead et al., 2010). There are however marked differences with dogs plasma OT concentration measurements from studies in which the plasma was not extracted (Handlin et al., 2011; Rehn et al., 2013); this discrepancy is to be expected since such discrepancies have already been extensively documented in the literature from human samples (Szeto et al., 2011; McCullough et al., 2013; Christensen et al., 2014) where higher OT concentrations in unextracted plasmas have been demonstrated to be due to the presence of non-OT immunoreactive products. In cats, the authors found only one study dealing with plasma OT concentrations in the species that reported a basal level between 20 and 30 pg/ml in solid-phase extracted samples and assayed with “in-house” EIA (Fieni et al., 2006). In three studies (Ginther and Beg, 2009, 2011, 2012) using horse samples and a very similar method of extraction and assay comprising a concentration factor of 13, Ginther and Beg found OT concentrations between 10 and 35 pg/ml, which are close to the values we found once the concentration factors have been applied. In cows, studies using RIA methods (Mačuhová et al., 2004; Belo and Bruckmaier, 2010) found basal levels of plasma OT between 1–4 and 3–6 pg/ml, which are similar to our results. Again, predictably, authors (Sutherland et al., 2012) using EIA methods with unextracted cow plasma exhibited OT concentrations 100-times higher than in the present study and studies using RIA. In sheep, basal plasma OT concentration was measured using RIA in the 5–10 pg/ml interval in extracted samples from ewes (Dwyer et al., 2004). In goats, reports using extracted samples and RIA or “in-house” EIA found basal plasma OT concentrations around 10 pg/ml (Payne and Cooke, 1998) or respectively in the interval of 14–47 pg/ml (Hernandez et al., 2002; Olsson and Högberg, 2009). Finally, basal plasma OT levels in pigs were described around 5–20 pg/ml in experiments using RIA following extraction (Castrén et al., 1993; Gilbert et al., 1996). This thorough comparison of our findings with the literature in every species supports the use of the method described and analytically validated here as a suitable means of measuring relevant OT measurements in the plasma of the domestic animal species involved in the study.

Of note, basal plasma OT levels among the seven studied species varied greatly. To the best of our knowledge, the reason for this species differences is not known, although our main assumption relates to the animals' size and the total volume of blood in each species: indeed, we experimentally noticed that the smaller the species is, the higher the basal levels of OT are. This would be interesting to further investigate it. Likewise, the question of sex differences in OT plasma levels is noteworthy when considering the increasing number of reports stating the sex importance in relation to the OT system (Neumann, 2008; Bakermans-Kranenburg and van IJzendoorn, 2013; Crockford et al., 2014; Alves et al., 2015), notably through the influence of steroid hormones, such as estrogen (Murakami et al., 2011; Acevedo-Rodriguez et al., 2015). Unfortunately, in this study, we did not specifically record the sexes of the enrolled animals as our purpose was to describe and validate a method which could be useful for numerous domestic species, regardless of the sex. Because the method has been especially validated on individuals from both sexes, we indeed think the method presented here could be helpful in studies investigating the sex effect on OT plasma levels. However, our recommendation would be to assay the samples from different animals' sexes on the same plate because the possible between-run variability found here may not permit to highlight small putative differences between sexes.

The procedure described in this study for assaying OT is mainly based on the procedure assessed by Szeto et al. (2011) for human plasma samples. These authors suggested that even extracted plasma contained non-OT immunoreactive products that could come from OT degradation. Thus, when we refer to OT, it should be understood as “oxytocin-like immunoreactive products.” Recently, some authors (Brandtzaeg et al., 2016) have described a new procedure to assay the total fraction of OT in plasma, in contrast with all previously published methods, including the one presented here, which aim to measure the free fraction of OT in plasma. They found that OT could bind to plasma proteins and thus be precipitated with them during the extraction procedure. In this regard, non-extracted protocols of OT assay may have partial immunoreactivity with bound OT, that could explain the method discrepancies. Treating the plasmas by reduction/alkylation could release OT molecules from plasma proteins, hence allowing higher amounts of OT to be measured, i.e., the total plasma OT fraction, notably in dog plasma. This could eliminate some drawbacks of the present method, such as the need to considerably increase the concentration of domestic animal samples, which requires drawing substantial amounts of blood. However, the biological activity of the bound fraction needs further clarification. Likewise, the interest of OT total fraction as a behavioral/emotional biomarker remains to be investigated.

## Conclusion

Method validation is a fastidious but critical step to providing reliable measurements and supporting strong scientific findings. The field of OT research is rapidly expanding. In human science, elegant advancements have been made to improve OT measurements and to establish standard, repeatable, reliable and validated methods to achieve them. Applying them to plasma samples of seven domestic animal species, the authors hope this study will help to provide reliable tools for measuring peripheral OT and supporting future studies on domestic animals in OT research fields.

## Author contributions

CB, AC, and PP wrote the manuscript and conceived the study. CB and CC analyzed the data, conceived, designed and performed the experiments. EC performed and supervised the blood sampling procedures in all species.

### Conflict of interest statement

The authors declare that the research was conducted in the absence of any commercial or financial relationships that could be construed as a potential conflict of interest.
